# 吉非替尼治疗*EGFR*基因突变状态不明的青年晚期肺腺癌疗效分析

**DOI:** 10.3779/j.issn.1009-3419.2014.05.06

**Published:** 2014-05-20

**Authors:** 雨桃 刘, 远凯 石, 兴胜 胡, 学志 郝, 峻岭 李, 子平 王, 燕 王, 宏羽 王, 湘茹 张, 燕 孙

**Affiliations:** 100021 北京，中国医学科学院北京协和医学院肿瘤医院内科，抗肿瘤分子靶向药物临床研究北京市重点实验室 Department of Medical Oncology, Cancer Institute/Hospital, Chinese Academy of Medical Sciences & Peking Union Medical College; Beijing Key Laboratory of Clinical Study on Anticancer Molecular Targeted Drugs, Beijing 100021, China

**Keywords:** 肺肿瘤, 腺癌, 吉非替尼, 青年, Lung neoplasms, Adenocarcinoma, Gefitinib, Young patient

## Abstract

**背景与目的:**

肺癌在青年患者中发病率较低，本研究旨在探讨吉非替尼治疗表皮生长因子受体酪氨酸激酶抑制剂（epidermal growth factor receptor, *EGFR*）基因突变状态不明青年晚期肺腺癌患者的疗效及生存情况的影响因素。

**方法:**

对2006年1月-2010年12月在中国医学科学院肿瘤医院接受吉非替尼治疗的55例*EGFR*基因突变状态不明的青年晚期肺腺癌患者的临床资料进行回顾性分析。

**结果:**

全组55例患者中位年龄41岁，吉非替尼治疗的客观有效率为43.6%，疾病控制率为90.9%。中位无进展生存期为9.0个月，是否存在脑转移对无进展生存期有明显影响（*P*=0.017）。中位总生存期为24.0个月，无吸烟史（*P*=0.028）及进展后是否接受后续治疗（*P* < 0.001）是明显改善总生存期的独立预后因素。

**结论:**

*EGFR*基因突变状态不明的青年晚期肺腺癌患者接受吉非替尼治疗与非年龄选择人群相比疗效相似。

## Abstract (en)

**Background and objective:**

Lung cancer in young patients (less or equal to 45 years) is relatively rare. We explored the efficacy and survival of Gefitinib for young patients with unknown epidermal growth factor receptor (*EGFR*) gene mutation of advanced lung adenocarcinoma.

**Methods:**

The clinical data of 55 young patients with unknown *EGFR* gene mutation in advanced lung adenocarcinoma referred to the Cancer Hospital & Institute, Chinese Academy of Medical Sciences from Jan 2006 through Dec 2010 were analyzed retrospectively.

**Results:**

Of 55 young patients enrolled, the median age was 41 years. The objective response rate and disease control rate were 43.6% and 90.9%, respectively.. The median progression-free survival (PFS) was 9.0 months. Among the factors analyzed, brain metastasis had significant effect on PFS (*P*=0.017). The median overall survival (OS) was 24.0 months. The independent prognostic factors to significantly improve OS included non-smoking history (*P*=0.028) and receiving other anti-cancer treatment after Gefitinib therapy (*P* < 0.001).

**Conclusion:**

The median PFS and OS of the young patients with Unknown EGFR gene mutation in advanced lung adenocarcinoma were similar with general population.

肺癌是全球最常见的恶性肿瘤，非小细胞肺癌（non-small cell lung cancer, NSCLC）占肺癌的80%-85%，大多数患者在疾病诊断时已发生远处转移，其5年生存率仅为15%左右^[1]^。美国流行病学调查发现肺癌患者初次就诊时的中位年龄是70岁，发病高峰为65岁-74岁^[2]^。NSCLC在青年患者中发病率较低^[3, 4]^，为1.2%-6.2%（40岁以下）^[3, 5, 6]^及1.4%-5.3%（45岁以下）^[2, 4]^。但亚洲及欧洲均有研究发现青年NSCLC发病率呈现逐年上升的趋势^[7, 8]^。来自中国台湾的数据显示，1991年-1999年，肺癌发病率的升高主要来自新发肺腺癌的增多，女性肺腺癌占肺癌的61.9%-77.8%，男性肺腺癌占33.8%-46.4%^[9]^。青年NSCLC的病理类型以腺癌为多见^[9-11]^，这也是与老年NSCLC在临床特征上的差异之一。

目前一些小分子的表皮生长因子受体酪氨酸激酶抑制剂（epidermal growth factor receptor-tyrosine kinase inhibitor, EGFR-TKI）已应用于晚期NSCLC治疗并取得了巨大的进展。吉非替尼（Gefitinib，Iressa，易瑞沙）是第一个应用于临床的EGFR-TKI，其不良反应较轻（主要是皮疹和腹泻），具有较好的耐受性^[12]^。尽管*EGFR*基因敏感突变已明确成为EGFR-TKI的疗效预测因子，但就我国肺癌诊治的临床现状来看，只有10%左右的NSCLC患者可以接受基因突变检测^[13]^，大部分患者的*EGFR*基因突变状态不明。而目前吉非替尼治疗*EGFR*基因突变状态不明的青年晚期肺腺癌的疗效分析尚缺乏大规模的前瞻性数据，本研究旨在通过回顾性分析，探讨青年晚期肺腺癌患者接受吉非替尼治疗的疗效影响因素，为临床治疗提供相关的数据参考。

## 1 资料与方法

### 1.1 临床资料

对2006年1月-2010年12月在中国医学科学院肿瘤医院接受吉非替尼治疗的55例*EGFR*基因突变状态不明的青年晚期肺腺癌患者的临床资料进行回顾性分析。入选患者要求开始吉非替尼治疗时的年龄≤45岁；细胞学或病理学证实为肺腺癌；临床分期为Ⅲb期或Ⅳ期（按照国际肺癌分期第7版NSCLC TNM临床分期标准）；吉非替尼治疗前接受过含铂化疗方案的治疗；接受过至少30天吉非替尼治疗，250 mg，每天1次，具有客观疗效评估依据，根据RECIST 1.1标准进行疗效评估；排除无细胞学或病理学证据的病例；排除临床分期不明或临床资料欠缺的病例。

### 1.2 疗效评价

吉非替尼治疗1月后进行首次计算机断层扫描（computed tomography, CT）复查，根据RECIST 1.1标准进行疗效评价，未进展的患者定期进行CT复查及随访。无进展生存期（progression-free survival, PFS）定义为自吉非替尼治疗开始至出现有客观证据证明疾病进展的时间。总生存期（overall survival, OS）定义为自吉非替尼治疗开始至任何原因导致死亡的时间。数据截止时仍存活的患者或未登记死亡日期的患者，以病历记录的最后日期作为截止日期。随访截止时间为2014年1月31日。

### 1.3 统计学分析

所有数据应用SPSS 16.0统计软件处理，对于不同亚组间疗效比较应用卡方检验；对生存时间分析应用*Kaplan-Meier*、*Log-rank*和*Cox*回归进行检验和多因素分析；以*P* < 0.05为差异具有统计学意义。

## 2 结果

### 2.1 一般资料

符合上述入选和排除标准的患者共55例，中位年龄41岁（28岁-45岁）。女性和男性患者分别为30例（54.5%）和25例（45.5%）。东部肿瘤协作组体能状态（East Cooperative Oncology Group performance status, ECOG PS）评分1分和2分的患者分别有52例（94.5%）和3例（5.5%）。11例有既往吸烟史的均为男性患者，另外14例男性患者及30例女性患者无吸烟史。临床分期为Ⅳ期的患者有48例（87.3%）。11例患者在吉非替尼治疗前存在脑转移，均接受过放射治疗，临床症状稳定或无相关临床症状。全组患者*EGFR*基因突变状态不明，所以均在吉非替尼治疗前接受过含铂化疗方案的治疗。吉非替尼作为二线和三线治疗的患者分别为38例（69.1%）及17例（30.9%）。

### 2.2 疗效评价

本组患者完全缓解（complete response, CR）0例，部分缓解（partial response, PR）24例（43.6%），稳定（stable disease, SD）26例（47.3%），进展（progressive disease, PD）5例（9.1%）。客观有效率（objective response rate, ORR）43.6%，疾病控制率（disease control rate, DCR）90.9%。。

### 2.3 生存分析

全组患者的中位PFS为9.0个月（1.0个月-53.0个月）（图 1）。对PFS进行*Kaplan-Meier*法分析后发现，是否存在脑转移对PFS的影响有统计学差异（图 2），不同性别、ECOG评分、是否有吸烟史、临床分期以及吉非替尼的治疗时机对PFS并无明显影响（表 1）（图 2）。*Cox*多因素分析显示，以上临床因素均不是影响PFS的独立因素。全组患者中位OS为24.0个月（4.0个月-63.0个月）。1年、2年生存率分别为78.2%和50.0%。本组患者吉非替尼治疗进展后，有8例（14.5%）患者未接受后续抗肿瘤治疗，其他患者继续接受了包括化疗、放疗、其他靶向治疗药物（包括EGFR-TKI）的治疗。对OS进行*Kaplan-Meier*法分析后发现，是否有吸烟史（*P*=0.017）和吉非替尼治疗进展后继续接受其他抗肿瘤治疗（*P* < 0.01）对OS的影响有统计学差异。*Cox*多因素分析显示，无吸烟史（*P*=0.028）以及吉非替尼治疗进展后继续接受其他抗肿瘤治疗（*P* < 0.001）是改善总生存期的独立预后因素，而不同年龄、性别、ECOG评分、临床分期、是否有脑转移、吉非替尼治疗时机对总生存期的影响无统计学差异。

**1:**
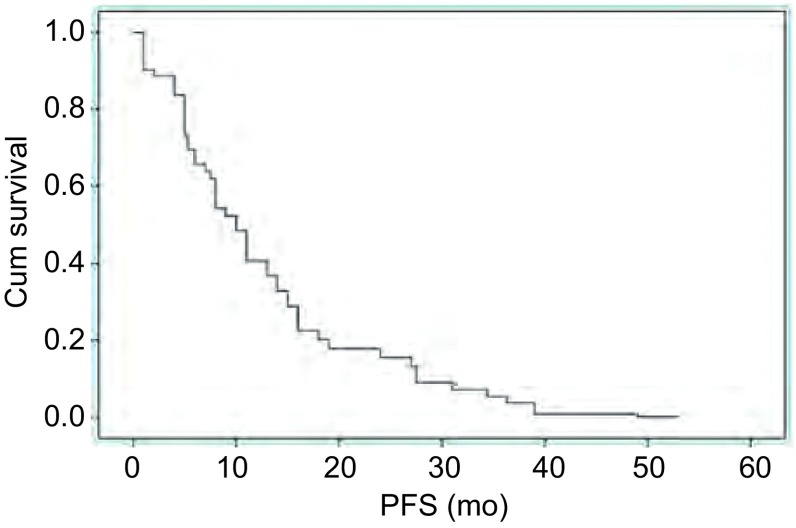
55例青年晚期肺腺癌患者PFS生存曲线 PFS survival curves of 55 young patients with advanced lung adenocarcinoma

**2:**
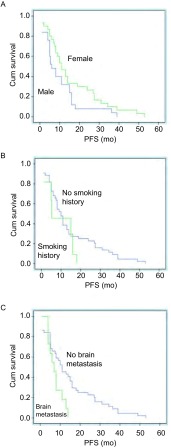
55例青年晚期肺腺癌患者的*Kaplan-Meier*生存曲线。A：不同性别的*Kaplan-Meier*生存曲线；B：不同吸烟状态的*Kaplan-Meier*生存曲线；C：不同脑转移状态的*Kaplan-Meier*生存曲线。 *Kaplan-Meier* survival curves of 55 young patients with advanced lung adenocarcinoma. A: *Kaplan-Meier* survival curves of sex; B: *Kaplan-Meier* survival curves of smoking history; C: *Kaplan-Meier* survival curves of brain metastasis.

**1:** 55例*EGFR*基因突变状态不明的青年晚期肺腺癌患者PFS PFS of 55 young patients with unknown *EGFR* gene mutation in advanced lung adenocarcinoma

Characteristics	Group	Median PFS (mo)	95%CI	*P*
Sex	Female	11.0	7.4-14.6	0.099
	Male	6.0	3.1-8.9	
Smoking history	No	10.0	7.9-12.1	0.241
	Yes	5.3	4.1-13.4	
ECOG PS	1	10.0	7.3-12.7	0.160
	2	9.0	7.4-10.6	
Stage	Ⅲb	11.0	0.0-23.8	0.116
	Ⅳ	8.0	5.6-10.4	
Brain metastasis	No	11.0	6.7-15.3	0.017
	Yes	7.0	4.6-9.4	
Treatment line	2^nd^ line	10.0	7.6-12.4	0.819
	3^rd^ line	6.0	0.8-11.2	
ECOG PS: Eastern Cooperative Oncology Group performance status; 95%CI : 95% confidence interval; PFS: progression-free survivial. EGFR: epidermal growth factor receptor.				

## 3 讨论

虽然肺癌的发病率及死亡率在全球均有逐年上升的趋势，但由于超过50%的晚期NSCLC患者就诊时年龄超过65岁^[14]^，发病高峰为65岁-74岁^[2]^，因此青年肺癌患者较少受到关注。近年来，探讨青年NSCLC患者的临床特征、治疗疗效及预后因素的几项研究^[15, 16]^结果也存在一些差异。但多项既往研究^[9-11]^发现青年NSCLC特别是青年女性NSCLC的病理类型以腺癌为多见。

目前，分子靶向药物特别是EGFR-TKI的出现，为晚期NSCLC特别是肺腺癌开辟了新的治疗途径。多项研究^[17-19]^显示具有某些特征的患者临床获益更加明显——如女性、无吸烟史、亚裔、腺癌等。本组55例青年晚期肺腺癌患者均未接受*EGFR*基因突变检测，但从临床特征来看54.5%为女性，80.0%无吸烟史，可见目前我院临床上接受吉非替尼治疗的青年晚期肺癌患者也多属于不吸烟、腺癌的“优势人群”。全组患者的ORR为43.6%，DCR为90.9%，高于吉非替尼用于非选择性人群二、三线治疗的IDEAL1和IDEAL2研究，其有效率为9%-19%，疾病控制率为68%-73%^[19-21]^，与入选均为优势人群的IPASS研究吉非替尼组43.0%的有效率相近^[22]^。

本研究发现是否存在脑转移对PFS的影响有明显差异，但不同性别、ECOG评分、是否有吸烟史、临床分期以及吉非替尼的治疗时机对PFS并无明显影响，考虑此结果与本组病例数较少有关。但女性和男性患者相比较，PFS（11.0个月*vs* 6.0个月）有改善的趋势，如继续扩大样本量，或能取得统计学差异。入选不吸烟或轻度吸烟、腺癌这一类选择性优势人群的IPASS研究显示使用吉非替尼一线治疗具有*EGFR*基因突变的晚期NSCLC较化疗能够明显延长PFS^[22]^，吉非替尼治疗组中位PFS为5.7个月，而*EGFR*基因突变亚组的中位PFS为9.5个月。入选亚裔肺腺癌患者的PIONEER研究显示，*EGFR*基因突变率可高达50%左右，其中不吸烟或女性亚组的突变率可达59.6%和60.6%^[23]^。目前尚无前瞻性临床研究报道45岁以下青年NSCLC患者的*EGFR*基因突变状态以及接受EGFR-TKI治疗的疗效。中国台湾有学者回顾性分析了144例45岁以下NSCLC患者的临床特征及治疗疗效，发现青年肺腺癌*EGFR*基因突变率为51.8%，但接受EGFR-TKI治疗的中位PFS和中位OS只有6.0个月和19.9个月，该学者认为可能青年晚期肺腺癌患者接受EGFR-TKI治疗的疗效差于非选择性人群^[11]^。本组患者中位PFS为9.0个月，优于IPASS研究吉非替尼组PFS，而与突变亚组相似，与上述台湾学者研究结果有所差异，考虑原因与本组入选患者为肺腺癌、80%不吸烟、54.5%女性，可能EGFR突变率较高有关。

全组患者中位OS为24.0个月，*Cox*多因素分析显示进展后继续接受其他抗肿瘤治疗能够明显延长总生存期，本组80%以上的患者在吉非替尼治疗进展后接受了后续治疗，提示我们青年晚期肺癌患者对治疗耐受性较好，进展后积极选择后续治疗或能进一步改善预后。

综上所述，*EGFR*基因突变状态不明的青年晚期肺腺癌患者二线或三线接受吉非替尼治疗与非年龄选择患者人群的既往数据相比较，临床疗效和生存期结果相近，不吸烟和进展后继续其他抗肿瘤治疗是影响生存的因素。如果患者的一般情况可耐受后续治疗，在EGFR-TKI治疗进展后应积极选择适当的后续治疗进一步改善预后。
